# Comparative Evaluation of Microshear Bond Strength and Microgap Formation of a New Self-Cure Bulk-Fill and a Light-Cure Composite Resin in Bonding to Dentin

**DOI:** 10.1155/ijod/9989327

**Published:** 2025-10-23

**Authors:** Seyedeh Maryam Tavangar, Mehrsima Ghavami-Lahiji, Reza Tayefeh Davalloo, Saman Maroufizadeh, Paria Shams

**Affiliations:** ^1^Department of Restorative Dentistry, School of Dentistry, Guilan University of Medical Sciences, Rasht, Iran; ^2^Department of Restorative Dentistry, Dental Sciences Research Center, School of Dentistry, Guilan University of Medical Sciences, Rasht, Iran; ^3^Department of Biostatistics and Epidemiology, School of Health, Guilan University of Medical Sciences, Rasht, Iran

**Keywords:** dental bonding, dental leakage, self-curing of dental resins

## Abstract

**Aim:**

This study aimed to compare the microshear bond strength (μSBS) and microgap formation of STELA self-cure composite and a light-cure composite when bonded to dentin.

**Material and Methods:**

A total of 45 bovine teeth were divided into three groups: (1) STELA composite with STELA primer (SS), (2) STELA composite with Clearfil SE Bond (S6), and (3) Luna light-cure composite with Clearfil SE Bond (L6). Each sample was bonded to dentin, subjected to artificial aging, and tested for μSBS using a universal testing machine with the wire loop technique. Failures were categorized as adhesive, cohesive, or mixed. Microgap formation at the dentin-composite interface was evaluated under a scanning electron microscope (SEM) at 1000× magnification. The average and maximum gap values were recorded. Data analysis was conducted using SPSS version 16, with statistical significance set at 0.05.

**Results:**

The L6 group exhibited significantly higher mean μSBS compared to both the S6 and SS groups (*p* < 0.001). Additionally, the SS group showed significantly higher bond strength than the S6 group (*p*=0.002). Adhesive failures were the most common in all groups, with cohesive failures in the composite being more frequent in the L6 group. However, the distribution of failure types did not differ significantly among groups (*p*=0.385). Microgaps in the L6 and SS groups were limited and comparable, although slightly higher in the SS group. The S6 group exhibited the largest maximum and mean microgaps, with gaps often extending across the entire interface.

**Conclusion:**

This study showed the highest bond strength in the light-cure composite, followed by STELA with its primer and then with Clearfil SE Bond. The largest microgap occurred in self-cure STELA with Clearfil SE Bond. STELA should be used with its specific primer, as Clearfil SE Bond is not a suitable substitute.

## 1. Introduction

Dental caries affect one-third of the global population, with dental composites, comprising 44% of treatments, as the most popular restorative material due to their physical and esthetic properties [1]. Composites are made of a polymer matrix, fillers, coupling agent, and initiator, curing either by light or chemical methods. Light-cure composites use light activation, while self-cure composites rely on chemical reactions [2, 3]. Light-cure composites are valued for their esthetics and strength, but may shrink during curing, creating microgaps at the composite–tooth interface. These gaps increase risks of bacterial infiltration, secondary caries, sensitivity, and failure of adhesion [4, 5]. Methods such as layering technique can help reduce shrinkage stress, improving restoration longevity, but they are time consuming [6].

Self-cure composites are regaining popularity in restorative dentistry due to their ease of use and quick application. Self-cure composites can be applied in a single layer, offering a quick and easy solution for restorative procedures. They also may offer better marginal adaptation and reduced microleakage than light-cure types. Their slower curing allows stress relief during bonding, while porosity from air bubbles further reduces shrinkage stress [7]. These composites generally perform well in strength and durability. However, their mechanical properties vary depending on their resin and filler content and are often lower than those of light-cure composites [8, 9].

The bond strength of self-cure composites can decrease with acidity levels in the oxygen-inhibited layer of some adhesive systems, as acidic monomers react with the amine components in self-cure composites, leading to poor polymerization at the interface [10, 11]. To prevent this, using three-step total-etch or two-step self-etch adhesives is recommended, as these adhesives add a protective resin layer that minimizes acidic monomers at the interface, reducing water absorption, hydrolysis, and bond degradation overtime. Simplified adhesives without this layer, like two-step total-etch or single-step self-etch adhesives, are less effective and may weaken bond strength with self-cure composites [12]. Studies also confirm the use of certain universal light-cure adhesives under self-cure composites. Manufacturers have introduced activators to address amine interference, while low-acidity adhesives (pH > 3) may not need them, though this is still debated [10, 13].

STELA (STELA Automix/Capsule, SDI Ltd., Australia) is a new self-cure composite with a special adhesive system (STELA primer) that does not require light activation. The primer, containing a catalyst, enhances polymerization at the interface, while fillers like strontium fluoroaluminosilicate and silica improve durability and bond strength without using BPA or hydroxyethyl methacrylate (HEMA)–based resin monomers. Based on the manufacturer's claims and relevant articles regarding the superior physical and mechanical properties of this composite compared to its counterparts, and due to the lack of sufficient studies on its characteristics such as bond strength and mechanical and physical properties, this composite was selected for the study [14–16]. Since the STELA primer is mostly sold in combination with the composite itself, once the primer in the packaging is used up, the remaining composite becomes unusable. If acceptable bond strength were achieved when using another adhesive instead of the STELA primer, it would be possible to recommend this adhesive as an alternative to the STELA primer.

This study aims to evaluate the microshear bond strength (μSBS) to dentin and the microgap formation at the interface of STELA self-cure bulk-fill composite and compare these properties with those of a light-cure composite following an artificial aging process simulating 6 months of natural aging. Additionally, the bond strength of STELA will be assessed when used with its dedicated primer versus a two-step self-etch adhesive.

The first hypothesis of this study is that the μSBS, microgap formation, and failure type in the STELA composite used with the STELA primer are not different from those in the same composite when used with the self-etch two-step adhesive. The second hypothesis of this study is that the μSBS, microgap formation, and failure type in the STELA composite used with the STELA primer do not differ from those in the light-cure composite used with the self-etch two-step adhesive. The third hypothesis of this study is that the μSBS, microgap formation, and failure type in the STELA composite used with the self-etch two-step adhesive do not differ from those in the light-cure composite using the same adhesive.

## 2. Materials and Methods

### 2.1. Preparation of Dental Samples

The samples were divided into three main groups (*n* = 15) based on the type of composite (STELA and Luna) and the primer or adhesive used (Clearfil SE Bond and STELA primer). For each group, 15 healthy bovine teeth were collected. The samples were stored for less than a month at 4°C temperature in distilled water with a neutral pH of around 7, after cleaning any tissue residues using a scaler and disinfectant solution (chloramine solution for 1 week).

The coronal part of the teeth was sectioned from the cervical region using a diamond disk and mounted in acrylic molds (Acroparars, Karaj, Iran). To prepare the samples, the teeth were grounded using an orthodontic trimmer under water cooling, leaving a flat dentin surface in the middle region. The dentin surface was then polished using a sequential grinding process with silicon carbide (SiC) papers of 320, 400, 600, and 800 grit, each applied for 30 s under a water stream, to create a smear layer on a smooth and desired surface.

Three study groups were: (1) STELA (Automix) self-cure composite with STELA primer (SS), (2) STELA self-cure composite with two-step self-etch Clearfil SE Bond (Kuraray Ltd., Japan) adhesive (S6), and (3) Luna light-cure composite (Luna, SDI Ltd., Australia) with two-step self-etch Clearfil SE Bond adhesive (L6; Table 1).

In each group, the primer or adhesive was applied to the dentin surface according to the manufacturer's instructions. For Clearfil SE Bond, the primer was applied to the surface using a disposable brush from the first bottle, as instructed. After waiting for 20 s, it was gently air-dried using an air syringe. Then, the adhesive from the second bottle was applied and evenly distributed using a gentle stream of air. Finally, light curing was performed for 10 s. The curing was done from an approximate distance of 2 mm using an LED light-curing unit (Bluedent, LED Smart, Bulgaria) with 1000 mW/cm^2^ intensity. For the STELA primer, the entire cavity was coated with the primer, then, left for 5 s, followed by 2–3 s of gentle air drying. No light curing was required.

In the subsequent step, transparent Tygon tubes (1 mm inner diameter and 4 mm height) were filled with the composites and positioned on the central dentin surface, using two layers for the Luna composite and one layer for the STELA composite. After removing any excess, the Luna composite samples were light-cured from above for 40 s, according to the manufacturer's instructions. For the STELA composite, a 4-min wait was observed as per the manufacturer's instructions, followed by an additional waiting period of up to 10 min to ensure complete setting before proceeding with further experimental steps.

The tube was then cut with a #15 scalpel blade and removed from the sample without any pressure.

### 2.2. Measurement of μSBS and Evaluation of Failure Types

After completing these steps, the samples were stored in distilled water until testing. Subsequently, they underwent 5000 cycles of thermocycling at temperatures ranging from 5 to 55°C using a thermocycling device (Nemo Mecatronica, Mashhad, Iran). μSBS was measured using a universal testing machine (SANTAM-20, Iran, Tehran) with the wire loop technique, at a thickness of 0.2 mm and a loading rate of 0.5 mm per minute. The maximum force applied at the moment of failure was recorded in megapascals.

The separated samples were examined under a stereomicroscope (EchoLab, DEVCO s.r.l, Italy) at a magnification of 40× to assess the failure modes. It is worth noting that all procedures were carried out under standard environmental conditions, at room temperature, and relative humidity.

Failure types were classified as follows:

  T1: Cohesive (more than 80% of the failure occurs in the dentin or composite).
  T2: Adhesive (more than 80% of the failures occur at the adhesive interface).
  T3: Mixed (a combination of adhesive and cohesive failures).

### 2.3. Microgap Evaluation

To assess internal adaptation and microgap formation, scanning electron microscopy was used (Mira/LMU, Tescan, Czech Republic). One sample from each group was prepared following the previous instructions and the primer and adhesive were applied to the exposed dentin surface as described earlier. Following the manufacturer's instructions, the Luna composite was light-cured for 40 s from above the samples, while the STELA composite was left to set for 4 min without light curing.

Each sample was then sectioned into mesial and distal halves using a 0.3 mm diamond disk with water cooling, in a buccolingual direction. One half of each sample was polished using 600 and then, 1200-grit paper discs. The samples were mounted in resin acrylic and polished for another 10 s. To remove debris from the gaps, the etching process was first carried out with 10% phosphoric acid for 5 s, followed by placing the samples in an ultrasonic cleaner for 10 min and finally gently air-drying them.

The samples were then mounted on aluminum stubs and sputter-coated with gold, before being examined under SEM. The samples were analyzed at 1000× magnification and the microgaps were measured using SEM software. For each sample, five distinct gap areas were identified using two reference points: one on the tooth and the other on the composite. The distance between these points was measured in micrometers and the average and maximum values were recorded for each sample.

### 2.4. Statistical Data Analysis

In this study, the values of continuous variables are presented as “mean (standard deviation)” and the values of categorical variables are shown as “frequency (percentage).” To assess the normality of the μSBS the Shapiro–Wilk test was used. The Levene's test was employed for the homogeneity of variances. For comparing the mean μSBS between different composites, one-way analysis of variance (ANOVA) and Tukey's post hoc test were applied. The chi-square test was used to compare the failure types among the composites. The data were analyzed using SPSS software version 16, with a significance level set at *p*  < 0.05. Additionally, GraphPad Prism version 8.0.1 was used to create the graphs.

### 2.5. Ethical Approval

This study was approved by the Ethics Committee of Guilan University of Medical Sciences under ethical code number IR.GUMS.REC.1403.352. All participants provided informed consent prior to sample collection.

## 3. Results and Discussion

### 3.1. Results

**Figure 1:**
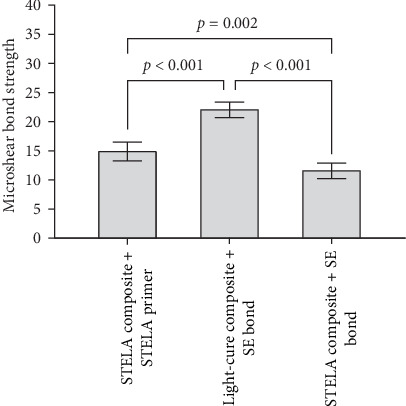
Comparison of microshear bond strength mean values in study groups. The values in the bar chart represent “mean values with 95% confidence interval.” The *p* value is based on one-way analysis of variance (ANOVA) and Tukey's post hoc test.

A one-way ANOVA and Tukey's post hoc test were used to compare the mean μSBS among the different groups. Before performing the one-way ANOVA, we tested the assumptions of normality and homogeneity of variances. Based on the Shapiro–Wilk test, the normality assumption was met for SS, L6, and S6 groups (*p*=0.398, *p*=0.139, and *p*=0.473, respectively). According to Levene's test, the homogeneity of variances was also met (*p*=0.426). As shown in Table 2 and Figure 1, there was a statistically significant difference in the mean μSBS among the three groups (*F*_(2, 46)_ = 69.06, *p* < 0.001, *η*^2^ = 0.750). The effect size, calculated using eta squared, was 0.000, which is considered to be large. Tukey's post hoc test results indicated that the mean μSBS of the L6 group was significantly higher than that of both the SS and S6 groups (*p* < 0.001 for both comparisons). Additionally, the mean μSBS of the SS group was significantly higher than that of the S6 group (*p*=0.002).

**Figure 2:**
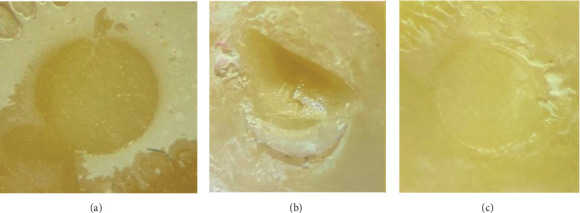
Different failure types under a stereomicroscope at 40× magnification. (a) adhesive, (b) mixed, and (c) cohesive.

**Figure 3:**
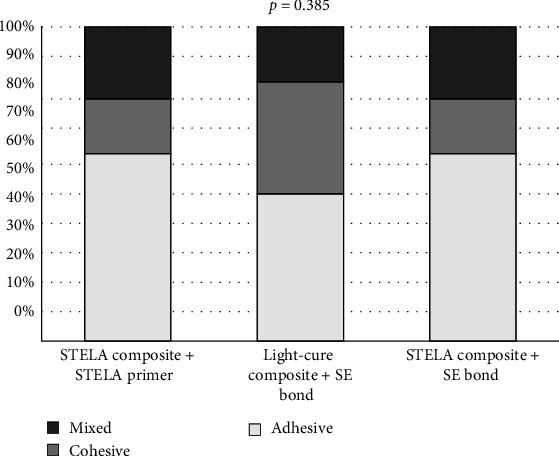
Comparison of failure types in study groups. The *p* value is based on the chi-square test.

A chi-square test was used to compare the distribution of failure types (Figure 2) among the different groups. As shown in Table 3 and Figure 3, there was no statistically significant difference in the distribution of failure types among the three groups (*χ*^2^_(4)_ = 4.16, *p*=0.385, Cramér's *V* = 0.158). The effect size, calculated using Cramér's *V*, was 0.158, which is considered to be small.

**Figure 4:**
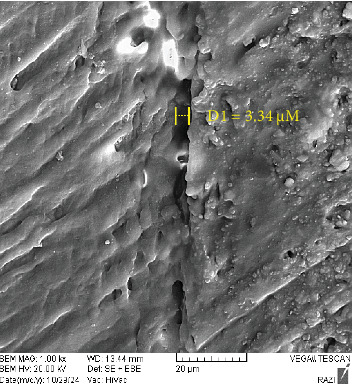
SEM image of STELA self-cure composite with STELA primer (the maximum microgap size is indicated in the image).

**Figure 5:**
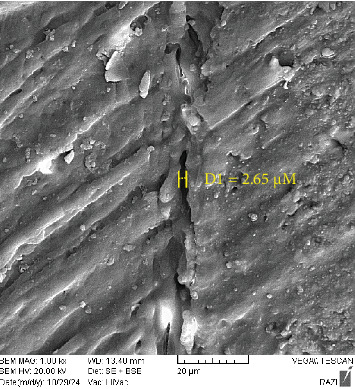
SEM image of Luna light-cure composite with Clearfil SE Bond (the maximum microgap size is indicated in the image).

**Figure 6:**
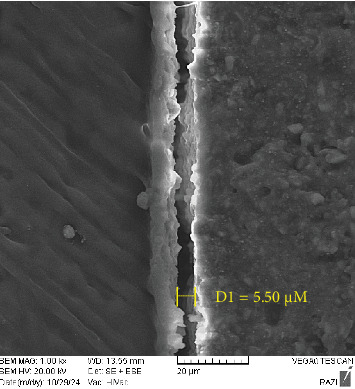
SEM image of STELA composite with Clearfil SE Bond (the maximum microgap size is indicated in the image).

As shown in Table 4, the amount microgap in the L6 and SS groups was limited and comparable, although the maximum and mean value were slightly larger in the SS group. The maximum and mean value of microgap in the S6 group were greater than in the other two groups, with the gap extending almost across the entire interface (Figures 4–6).

### 3.2. Discussion

Today, resin composites have largely replaced materials like amalgam in dentistry, owing to their excellent esthetic and mechanical properties. Resin composites are now the preferred material for many dental restorations, surpassing amalgam and glass ionomer cements. Their versatility allows them to be used for various applications, including restorations, fissure sealants, inlays, crowns, temporary restorations, and as luting agents for cementation [1, 17].

Upon exposure to light in light-cure composites, the initiator components start a polymerization process that hardens the material. However, these composites undergo volumetric shrinkage during polymerization, which can create tensile stresses at the interface between the composite and tooth [18]. This stress can cause gaps to form, potentially resulting in posttreatment sensitivity, marginal discoloration, and secondary caries. To minimize these issues, methods such as the layering technique is recommended [5].

While light-cure composites are more commonly used, self-cure composites still play an important role in dentistry. They cure without light exposure and typically experience less shrinkage stress due to slower polymerization, allowing more time to bond with the tooth and reducing issues like microleakage. Self-cure composites are also advantageous in time-sensitive situations since they do not require layering or light curing [8]. In self-cure composites, air bubbles may be introduced during mixing. However, these air bubbles can act as buffers, helping to reduce contraction stress by providing a larger surface area within the composite material. Nevertheless, the presence of these porosities can result in decreased physical and mechanical properties [19, 20]. Self-cure composites generally demonstrate optical and mechanical properties that are comparable to or even superior to materials like amalgam. However, they typically fall short when compared to light-cure composites in these areas. These properties can vary significantly between different self-cure composites, mainly due to differences in resin matrix composition and filler types [14].

The current study aimed to compare the μSBS and microgap formation of STELA self-cure composite (with STELA primer and a two-step self-etch adhesive) to a light-cure composite when bonded to dentin. STELA, introduced in 2023, has been reported to have superior properties over other self-cure composites and some bulk-fill light-cure composites. With a unique bonding process, STELA is designed for ease and speed, though more research is needed to fully validate its clinical benefits.

Thadathil Varghese et al. [14] compared the physical and mechanical properties of dual-cure and self-cure composites, including STELA Capsule and STELA Automix. Their findings largely favored STELA composite, with STELA demonstrating higher flexural strength, flexural modulus, compressive strength, and surface hardness. These superior qualities were attributed to the composition of STELA's filler content [14].

Previous studies have shown that self-cure composites tend to have lower bond strength than light-cure composites. However, recent research suggests that some self-cure composites, including STELA, may demonstrate comparable bond strengths [11, 16].

μSBS test, known for its reliability due to homogenous stress distribution across a small bond area, was conducted in this study using the wire loop method. This method has been noted by Foong et al. [21] for being easier and more reliable than the blade technique, while Barga et al. [22] reported that using a chisel for load application could cause stress concentration and lower bond strength than the wire loop technique.

μSBS analysis in our study revealed that the mean bond strength of the L6 group was significantly higher than that of both the SS and S6 groups. In a study by Pires et al. [16], SEM analysis of the composite surface following adhesive failure revealed dentin tubules occluded by smear layer and unexposed collagen fibrils, suggesting that the lower bond strength of STELA might stem from insufficient primer penetration into tubules, limited surface conditioning, or lack of collagen fibril exposure.

In the previously mentioned study, STELA demonstrated the highest immediate μSBS in an etch-and-rinse condition compared to light-cure bulk-fill and conventional light-cure composites. However, all materials showed a significant decrease in bond strength after long-term storage, with the conventional light-cure composite exhibiting the best bond strength postaging. It is noteworthy that the use of etching for STELA was not recommended by the manufacturer. In cases where both chemical and micromechanical bonding are desired, maintaining hydroxyapatite is emphasized for the effectiveness of 10-MDP and ionglass monomers. When used as recommended (without etching), STELA's bond strength after 12 months of aging was lower than that of conventional light-cure composites, although the difference was not statistically significant [16]. The discrepancy in bond strength with our study may be due to differences in the light-cure composites, adhesive systems, and bond strength testing methods used.

Studies suggest that microshear bond test results in lower stress concentrations than macro tests, generally leading to adhesive rather than cohesive failure patterns [23, 24]. In the current study, adhesive failure was the predominant failure type across all groups, underscoring the reliability of microshear bond test conducted. The L6 group exhibited a higher rate of cohesive failures, which implies a stronger bond in this group compared to others. This observation challenges the claim that STELA's bond strength matches that of the light-cure composites. Given that the mean μSBS for STELA with its primer in this study was 14.89 MPa, further research is essential to confirm its clinical suitability.

Gaps in composite–dentin interface may arise from various factors, including insufficient bonding, polymerization shrinkage, adhesive degradation, aging-induced fatigue, disparities in the thermal expansion coefficients of substrates and restorative materials, the finishing and polishing process, and voids due to incomplete material coverage [25].

In the present study, SEM was used at 1000× magnification for microgap evaluation. Both the L6 and SS groups displayed limited and comparable gap formation, though the mean and maximum gap dimensions were slightly higher in the SS group. These findings contradict the claim that STELA has fewer gaps than light-cure composites.

In their research, Pires et al. [16] observed that STELA exhibited superior tooth bonding with fewer gaps and better interfacial adaptation compared to bulk-fill light-cure composites. Their study utilized confocal microscopy for gap analysis, showing no gaps in the etch-and-rinse mode for conventional light-cure composites and limited gaps in the self-etch mode, with nearly no gaps for STELA in either mode [16].

The difference in the observation of microgaps in our study compared to the Pires study may be due to the techniques used for observing the microgaps. In the Pires study, the gaps were examined by fluorescent dye penetration and evaluated under a confocal microscope. However, in our study, direct observation of the gaps was conducted under an SEM at higher magnification. The bond strength results obtained in our study may also support these findings. Additionally, the differences in sample preparation between the two studies could have influenced the variation in results. However, given the limited SEM sample size in our study, more extensive research on microgap and microleakage formation in STELA is necessary.

One of the primary issues with the use of self-cure composites is their incompatibility with certain adhesives containing acidic resin monomers [26, 27]. This problem is particularly evident in two-step total-etch adhesives and one-step self-etch adhesives, where, first, the high concentration of acidic monomers in the adhesives reacts with the amine components of the catalyst or initiator in self-cure composites. These reactions can lead to incomplete polymerization and a reduction in bond strength between the composite and the tooth [28, 29]. Second, the concentration of acidic, ionic, and hydrophilic monomers in these adhesives increases their permeability, which may contribute to a decrease in bond strength and an increased incidence of adhesive failure in these composites [30, 31]. To mitigate these issues, three-step total-etch or two-step self-etch adhesives are recommended. In these systems, an additional resin layer is applied, acting as a protective barrier that reduces the negative effects of acidic and hydrophilic monomers. This approach enhances long-term bond durability, strength, and protection against gradual degradation. In contrast, adhesives with fewer steps, such as two-step total-etch or one-step self-etch systems, tend to show reduced compatibility with self-cure composites [28, 29]. King et al. [12] demonstrated that applying a hydrophobic resin layer over one-step self-cure light-cure adhesives (7th generation) effectively converts them into two-step self-cure adhesives (6th generation), enabling better compatibility with self-cure composites and yielding improved bond strength.

The use of certain universal light-cure adhesives beneath self-cure composites has also been validated by studies. To address the issue of amine interference with acidic monomers in these adhesives, manufacturers have developed a separate activator bottle. This bottle contains substances such as sodium toluenesulfonate and ethanol, which, when combined with the adhesive, resolve the bonding interference. Universal adhesives with relatively low acidity (pH > 3) likely do not require an activator, although this remains a topic of ongoing discussion in the literature [10, 13].

STELA primer, however, is mainly available as part of the STELA composite, creating a limitation if the primer is depleted. If an adhesive could achieve comparable bonding to STELA's specific primer, it would provide a practical alternative for dental professionals. However, the present study's μSBS test results indicated that the mean bond strength for the S6 group was significantly lower than that of both the L6 and SS groups. Additionally, SEM images showed wider and more frequent microgaps in this group, suggesting incomplete dentin bonding across the surface.

As mentioned before, in self-cure composites, the amine component (aromatic tertiary amines) typically interferes with the acidic monomers in adhesives, negatively affecting polymerization [32]. However, according to the manufacturer, STELA is an amine-free composite, and its initiator is a ketone (methyl ethyl ketone). However, some components of the material, such as its coinitiator, are not disclosed and may interact with the self-etch bonding system used in this study. A 10-MDP has both acidic properties and the ability to form chemical bonds. It can create stable bonds with hydroxyapatite and interact with the fillers in STELA, potentially altering the expected ion exchange and polymerization process in STELA. While 10-MDP is listed in STELA's primer formulation, its concentration is unspecified and could differ from that in Clearfil SE Bond, leading to different results. The HEMA in Clearfil SE Bond acts as a plasticizer [33]. Although HEMA improves wetting and adhesion, it may interfere with polymerization. MDP interacts with hydroxyapatite to form stable MDP-Ca salts (nano-layering), enhancing adhesion. However, previous studies concluded that HEMA inhibits nano-layering by 10-MDP, which can increase microgap and microleakage while reducing bond strength [34, 35]. Given that both 10-MDP and HEMA (in SE bond) are hydrophilic, their interaction with STELA's components may lead to increased water uptake at the bond interface. This could promote hydrolytic degradation overtime, further impacting bond durability.

Therefore, this study does not support substituting the STELA primer with a two-step self-etch adhesive like Clearfil SE Bond.

All curing procedures were conducted at room temperature (~23–24°C), which was consistently monitored and maintained throughout the experiment. This temperature range aligns with standard laboratory conditions and is within the typical range recommended for polymerization of self-curing dental composites.

The samples in this study were stored in distilled water for less than 1 month at 4°C temperature and disinfected with chloramine solution prior to testing. Literature indicates that such storage conditions, including short-term aging, do not significantly affect dentin microtensile bond strength [36]. Moreover, chloramine has been shown not to alter the hardness, elastic modulus, or morphology of the resin–dentin interface. However, some studies suggest it may interfere with the interaction between adhesive resin and dentin in chemical-cured adhesives, potentially influencing bond performance [37]. Given its water solubility, chloramine likely dissipates during storage, particularly when teeth are immersed in distilled water, minimizing its potential long-term effect on bonding. Nonetheless, this factor should be considered in interpreting results and in the design of future studies.

Given the limited number of samples analyzed with SEM in this study, further research is recommended to investigate the microgap and microleakage of this composite. According to the manufacturer, the STELA primer acts as a catalyst for its composite. Due to the small amount of samples in the μSBS test, the results may have been influenced by sample size. It is suggested that future studies explore different bond strength tests, such as microtensile or macrobond strength tests, for a thorough evaluation.

## 4. Conclusion

In this study, the highest μSBS was observed with light-cure composites. Following this, STELA composite with STELA primer demonstrated higher bond strength than STELA composite with Clearfil SE Bond. The analysis of failure modes within the groups also confirmed the bond strength test results.

Given the limitations of this study, the microgap in the self-cure STELA composite group with STELA primer was greater than that of the light-cure composite. The highest microgap was observed in the self-cure STELA composite group with Clearfil SE Bond.

This study did not support the use of the two-step self-etch adhesive, Clearfil SE Bond as a substitute for the STELA primer. It is recommended that STELA be used with its dedicated primer.

## Figures and Tables

**Table 1:** Description of materials used in the present study.

Group	Manufacturer	Composition
STELA automix	SDI Ltd., Australia	Methacrylates, urethane dimethacylate, glycerol dimethacrylate, 10-methacryloyloxydecyl dihydrogen phosphate (10-MDP), barium–alumino-borosilicate glass, fluoroaluminosilicate glass, ytterbium trifluoride, silicon dioxide (hydrophobic fumed silica) sodium persulphate, calcium aluminate Initiators, stabilizers, pigments
STELA primer	SDI Ltd., Australia	Methacrylates (including 10-MDP), methyl ethyl ketone (MEK), water, initiators, stabilizers
Luna composite	SDI Ltd., Australia	Diurethane dimethacrylate (DUDMA), triethyleneglycol dimethacrylate (TEGDMA), 2,2-bis[4-(2-methacryloxy)ethoxy)phenyl]propane, initiators, stabilizers
Clearfil SE Bond	Kuraray Ltd., Japan	Primer: 10-MDP, 2-hydroxyethyl methacrylate (HEMA), hydrophilic aliphatic dimethacrylate, dl-camphorquinone, N,N-diethanol-p-toluidine, water. Bond: 10-MDP, bisphenol A, diglycidylmethacrylate (Bis-GMA), 2-hydroxyethyl, HEMA, hydrophobic aliphatic dimethacrylate, dl-camphorquinone, N,N-diethanol-p-toluidine, colloidal silica

**Table 2:** Microshear bond strength in the study groups.

Group	Mean (SD) of the μSBS (MPa)
STELA primer + STELA composite	14.89 (3.00)
SE bond + light-cure composite	22.4 (2.46)
SE bond + STELA composite	11.57 (2.36)

**Table 3:** Distribution of failure types (percentage) in study groups.

Group	Mixed	Cohesive	Adhesive
STELA primer + STELA composite	17.9	17.9	64.3
SE bond + light-cure composite	14.3	35.7	50.0
SE bond + STELA composite	22.2	14.8	63.0

**Table 4:** Microgap values in study groups.

Group	Microgap values (µm)	
	Mean	Maximum
STELA primer + STELA composite	3.22	3.34
SE bond + light-cure composite	2.22	2.65
SE bond + STELA composite	4.72	5.50

## Data Availability

The data that support the findings of this study are available from the corresponding author upon reasonable request.
